# Hippocampus-centred grey matter covariance networks predict the development and reversion of mild cognitive impairment

**DOI:** 10.1186/s13195-023-01167-z

**Published:** 2023-02-02

**Authors:** Mingxi Dang, Caishui Yang, Kewei Chen, Peng Lu, He Li, Zhanjun Zhang, for the Beijing Aging Brain Rejuvenation Initiative, for the Alzheimer’s Disease Neuroimaging Initiative

**Affiliations:** 1 State Key Laboratory of Cognitive Neuroscience and Learning, Faculty of Psychology, Beijing Normal University, Beijing, 100875 China; 2 School of Systems Science, Beijing Normal University, Beijing, 100875 China; 3 Banner Alzheimer’s Institute, Phoenix, AZ 85006 USA; 4 Institute of Basic Research in Clinical Medicine, China Academy of Chinese Medical Sciences, Beijing, 100700 China

**Keywords:** Mild cognitive impairment, Covariance network, Hippocampus, Partial least square analyses, Random forest, Default network, Frontoparietal network

## Abstract

**Background:**

Mild cognitive impairment (MCI) has been thought of as the transitional stage between normal ageing and Alzheimer’s disease, involving substantial changes in brain grey matter structures. As most previous studies have focused on single regions (e.g. the hippocampus) and their changes during MCI development and reversion, the relationship between grey matter covariance among distributed brain regions and clinical development and reversion of MCI remains unclear.

**Methods:**

With samples from two independent studies (155 from the Beijing Aging Brain Rejuvenation Initiative and 286 from the Alzheimer’s Disease Neuroimaging Initiative), grey matter covariance of default, frontoparietal, and hippocampal networks were identified by seed-based partial least square analyses, and random forest models were applied to predict the progression from normal cognition to MCI (N-t-M) and the reversion from MCI to normal cognition (M-t-N).

**Results:**

With varying degrees, the grey matter covariance in the three networks could predict N-t-M progression (AUC = 0.692–0.792) and M-t-N reversion (AUC = 0.701–0.809). Further analyses indicated that the hippocampus has emerged as an important region in reversion prediction within all three brain networks, and even though the hippocampus itself could predict the clinical reversion of M-t-N, the grey matter covariance showed higher prediction accuracy for early progression of N-t-M.

**Conclusions:**

Our findings are the first to report grey matter covariance changes in MCI development and reversion and highlight the necessity of including grey matter covariance changes along with hippocampal degeneration in the early detection of MCI and Alzheimer’s disease.

**Supplementary Information:**

The online version contains supplementary material available at 10.1186/s13195-023-01167-z.

## Background

Mild cognitive impairment (MCI) is considered to be the transitional stage between normal ageing and clinical dementia [1], and patients with MCI have substantial increases in the risk of progression to dementia [2]. With the many clinical treatment failures for Alzheimer’s disease (AD) [3, 4], MCI has gradually become regarded as a critical time window for disease treatment and intervention [5–8].

Over the past few decades, much research has focused on the clinical and cognitive characteristics of MCI, and these studies have found that MCI is a heterogeneous at-risk state that does not necessarily lead to AD [9, 10]. Furthermore, studies with longitudinal design also found that clinically defined MCI was not a stable population [11–14]. Although most studies have focused on the progression of MCI to AD, some have found that up to 24 to 50% of MCI individuals would revert to normal cognition (NC) status [15, 16]. This evidence suggests the need to explore the mechanisms underlying MCI reversion and to identify the key biomarkers based on longitudinal clinical diagnosis.

With applications of neuroimaging methods like magnetic resonance imaging (MRI), previous studies have reported structural changes in grey matter (GM) in patients with MCI. For example, several studies have identified significant reductions in local GM volume in the hippocampus, entorhinal cortex, medial temporal lobe, insula, and thalamus in patients with MCI compared to cognitively normal older adults [17–19]. By contrast, as far as we know, limited studies have explored the brain structural basis of MCI reversion, and available evidence indicated that MCI individuals who reverted to NC have larger hippocampal and amygdala volumes than those who maintain stable MCI over the same time period [20, 21].

Furthermore, recent studies have also found that in a series of neurodegenerative diseases related to AD, patients have not only represented local GM degeneration, but also showed loss of synchronous GM changes among distributed brain regions, a phenomenon known as structural covariance [22], that is, interindividual differences in the structure of brain regions often covary with interindividual differences in other brain regions [23]. Compared with healthy individuals, AD patients have a significant decrease in the structural association between the entorhinal cortex and the medial prefrontal cortex, the posterior cingulate cortex, the inferior orbitofrontal gyrus, the right superior parietal lobule, and the left superior occipital gyrus [24, 25], which are key regions of the default network (DMN) and the frontoparietal network (FPN). In addition, another study found that decreased structural covariance in the frontotemporal regions and other regions was associated with cognitive decline among MCI patients [26]. However, to our knowledge, no evidence has focused on how the structural covariance network is related to the development and reversion of MCI.

In the current research, we aimed to (1) focus earlier on the GM covariance characteristics of NC individuals who progressed to MCI (N-t-M progression), (2) explore the GM covariance characteristics of MCI patients who reverted to the NC state (M-t-N reversion), and (3) validate the results using two independent longitudinal samples. Based on previous evidence, we hypothesized that reversed MCI patients presented a stronger covariance pattern of brain structure than non-reversed MCI patients, and progressive NC individuals showed a loss of structural covariance compared with NC individuals who maintained normal clinical status. Our study would assess the feasibility of using MRI biomarkers as a way to predict the development and reversion of MCI and provide more accurate biomarkers for the early diagnosis and effective intervention for MCI.

## Methods

### Sample characteristics

The participants were from the Beijing Aging Brain Rejuvenation Initiative (BABRI) [27] and the Alzheimer’s Disease Neuroimaging Initiative (ADNI) [28]. All participants were classified as NC or MCI at baseline using the Petersen criterion [29]. Briefly, the diagnostic criteria for MCI included subjective memory complaints, impairment in at least one cognitive domain (1.5 standard deviations or more), relatively preserved general cognitive function, and intact ability to perform activities of daily living. The criteria for normal cognition were no cognitive complaints, a Mini-Mental State Examination (MMSE) score of no less than 24, and being able to perform the normal activities of daily life. A total of 155 eligible participants from the BABRI and a total of 286 eligible participants from the ADNI were included in this study.

All participants from both cohorts underwent baseline MRI scans and had at least one clinical diagnosis during follow-ups. They were divided into four groups based on their clinical diagnosis at baseline and follow-ups: stable NC (sNC, NC at baseline and maintained cognitively normal until the last visit), progressed NC (pNC, NC at baseline, progressed to MCI during follow-ups, and maintained cognitively impaired until the last visit), non-reversed MCI (non-rMCI, MCI at baseline and maintained cognitive impairment or progressed to AD until the last visit), and reversed MCI (rMCI, MCI at baseline, reverted to NC during follow-up and maintained cognitively normal until the last visit). Participants with multiple (> 2) state fluctuations between NC and MCI were excluded.

### MRI data acquisition and processing

High-resolution T1-weighted MRI data were collected from each participant from the BABRI and ADNI using either 1.5-T scanners (participants from ADNI-1) or 3-T scanners (participants from BABRI and ADNI-GO&2); the acquisition parameters for each study have been published previously [27, 30–32]. Only baseline T1-weighted MRI data were used in the current study.

All T1-weighted images were preprocessed using the Computational Anatomy Toolbox (CAT12, http://dbm.neuro.uni-jena.de/cat12/) implemented in MATLAB (R2015a). First, the raw image was spatially registered to the tissue probability maps and then segmented into GM, white matter, and cerebrospinal fluid. Subsequently, we smoothed the GM image with a Gaussian kernel of 8 mm full-width-half-maximum (FWHW). Moreover, we assessed the processed image quality by visual inspection and the weighted average image quality index using the quality assurance (QA) framework in CAT12, and only participants with a QA better than C were included.

### Construction of the structural covariance network

As cognitive impairment might cause changes in the correlation between seeds and other brain regions [33], we established standard structural covariance networks based on 65 sNC from the BABRI and 47 sNC from the ADNI (“Template sNC” in Table 1).

**Table 1:** Demographic information of the BABRI and ADNI samples

	Template sNC	sNC	pNC	***T***/***χ***^**2**^	***p***	Non-rMCI	rMCI	***T***/***χ***^**2**^	***p***
**BABRI sample (*N *= 155)**									
No. of participants	65	28	18	–	–	20	24	–	–
Age, y	64.0 ± 6.8	65.8 ± 5.0	66.2 ± 5.8	− 0.25	0.804	68.0 ± 6.8	63.5 ± 6.4	2.253	**0.030**
Male, %	31 (47.7%)	13 (46.4%)	7 (38.9%)	0.253	0.615	11 (55%)	10 (41.7%)	0.777	0.378
Education, y	11.4 ± 2.9	11.8 ± 2.8	10.9 ± 3.4	0.963	0.341	11.5 ± 4.1	10.1 ± 2.7	1.249	0.221
MMSE	28.0 ± 1.6	28.5 ± 1.1	26.6 ± 1.8	17.563	**< 0.001**	25.3 ± 2.8	27.1 ± 1.6	3.585	0.066
**ADNI sample (*N *= 286)**									
No. Participants	47	47	54	–	–	65	73	–	–
Age, y	69.3 ± 4.5	73.9 ± 4.3	73.8 ± 3.7	0.134	0.894	74.2 ± 7.7	70.1 ± 8.3	1.663	0.099
Male, %	23 (48.9%)	23 (48.9%)	26 (48.1%)	0.006	0.937	34 (52.3%)	40 (54.8%)	0.086	0.770
Education, y	16.5 ± 2.7	15.7 ± 2.9	16.3 ± 2.4	− 1.204	0.231	16.0 ± 2.8	16.7 ± 2.4	− 1.477	0.142
APOE4 carrier status, %				4.478	0.107			14.331	**0.001**
APOE4 heterozygotes	17 (35.4%)	8 (17.0%)	19 (35.2%)	–	–	24 (36.9%)	27 (37.0%)	–	–
APOE4 homozygotes	1 (2.1%)	2 (4.3%)	1 (1.9%)	–	–	15 (23.1%)	2 (2.7%)	–	–
MMSE	29.1 ± 1.1	29.2 ± 0.9	28.9 ± 1.2	1.255	0.213	27.0 ± 1.6	28.6 ± 1.5	− 5.911	**< 0.001**

“Template sNC” is used only to establish a structural covariance network, independent of the sNC participating in statistical and predictive analysis

*Abbreviations*: *sNC* stable normal cognition, *pNC* progressive normal cognition, *Non-rMCI* non-reversed mild cognitive impairment, *rMCI* reversed mild cognitive impairment, *y* years, *N* number of participants

**Fig. 1:**
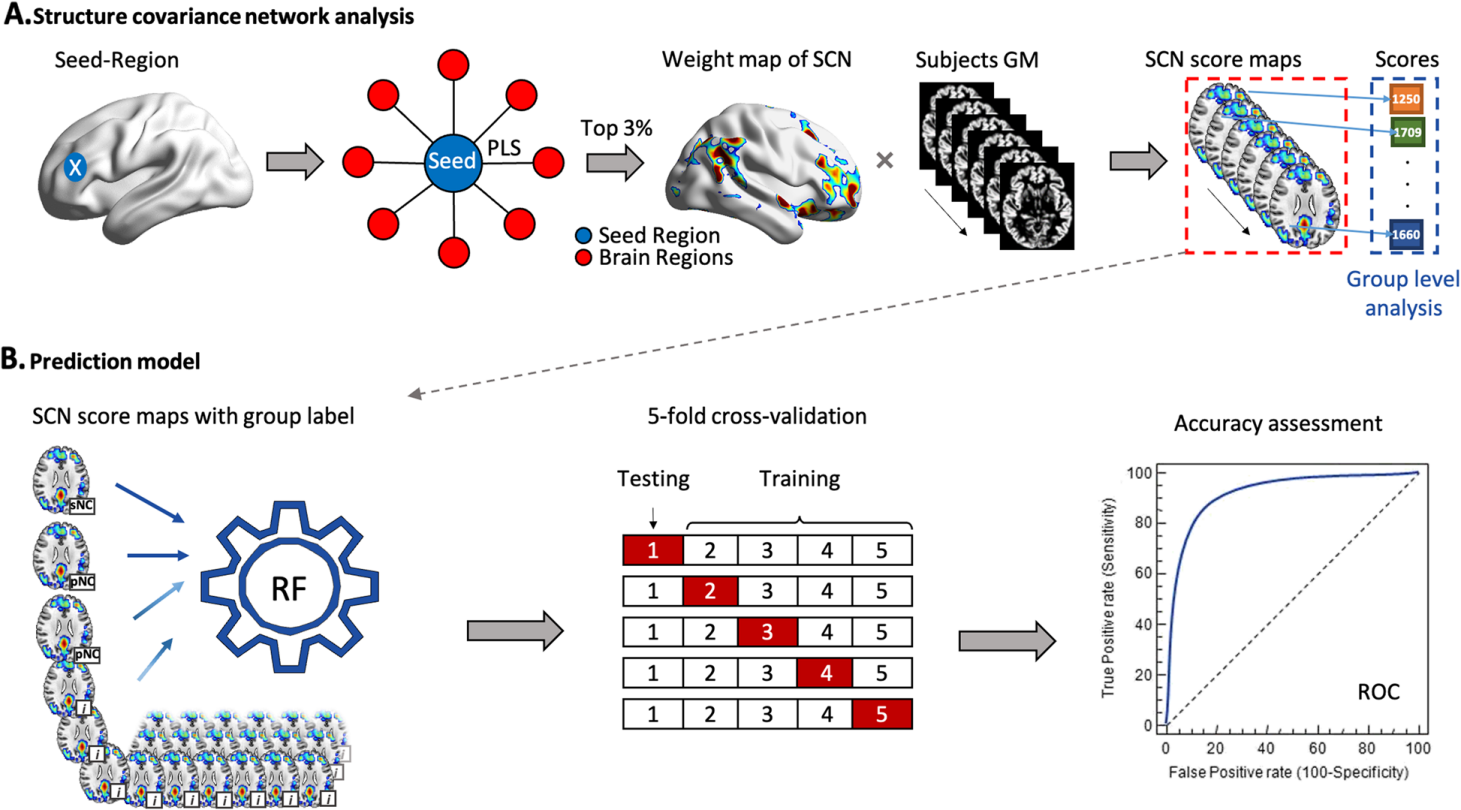
Schematic illustration of the structural covariance network and prediction model. **A** PLS regression was first conducted between the selected seed region and the remaining voxels throughout the whole brain, and the top 3% of voxels with the largest correlation (weight) with the seed region were retained to build a grey matter structural covariance network. Then, the structural covariance network score of each individual was calculated. **B** The prediction models of RF were established based on the baseline score maps of the covariance network. *Abbreviations*: SCN, structural covariance network; PLS, partial least squares; RF, random forest; sNC, stable normal cognition; pNC, progressive normal cognition; ROC, receiver operating characteristic curves

Seed-based partial least squares (seed-PLS) (PLSgui version 5.07 run on MATLAB 2015a) were used to construct the structural covariance network [34]. Seed-PLS is a data-driven multivariate statistical technique that reveals the covariance patterns of the GM structure throughout the brain and is methodologically applicable to large-scale structural covariance networks [35]. Briefly, we first identified the seed regions of each network and then carried out PLS regression with the GM density of the voxels in the GM map to obtain the voxel group with the strongest correlation with the seed regions (Fig. 1A). The between-participant correlation matrix of the structural integrity between the seed and the other voxels in the whole brain is decomposed into latent variables (LVs) that can identify patterns of structural correlation. The significance of the LVs was determined by 1000 non-parametric permutation tests using non-replacement resampling. The robustness and reliability of each voxel’s contribution to the LV were provided by a bootstrap that resampled the data 1000 times with replacement to estimate the standard error of the weight of each voxel on the LV. A bootstrap ratio (BSR), calculated as the ratio of each weight to its standard error and the threshold, was set to the top 3% of reliable voxels for display purposes and the calculation of the subsequent covariance network scores.

Finally, we calculated a score map of the structural covariance network for each participant, which is mathematically expressed as the dot product of the GM density in each individual’s preprocessed image and the corresponding voxel significance (i.e. weight) in the spatial pattern derived from the threshold PLS group result image (Fig. 1A). Then, we added up the scores of each voxel to obtain the composite score of each participant to represent the structural integrity of the GM. Pearson correlation is used to investigate the relationship between the composite score of the structural covariance network and the GM density of the hippocampus, a region well known to be affected in AD [21]. In the subsequent analyses, the composite scores and the score maps of the structural covariance network were used for intergroup analyses and individual-level prediction, respectively.

In this study, we focused on the DMN, FPN, and hippocampal network (HN), which have been reported to be the most sensitive to cognitive impairment and ageing [36–38]. The seed regions used to construct each network were defined based on a previous human brain atlas: for the DMN, the posterior inferior parietal lobule (*X* = − 41, *Y* = − 60, *Z* = 29) and posterior cingulate cortex (*X* = − 7, *Y* = − 52, *Z* = 26) [35]; for the FPN, the left anterior prefrontal cortex (*X* = − 36, *Y* = 57, *Z* = 9) and right anterior prefrontal cortex (*X* = 34, *Y* = 52, *Z* = 10) [39]; and for the HN, the left hippocampal cortex (*X* = − 25, *Y* = − 21, *Z* = − 10) and right hippocampal cortex (*X* =29, *Y* = − 20, *Z* = − 10).

### Statistical analyses

Differences in the demographic information and vascular risk factors between the sNC and pNC groups and between the non-rMCI and rMCI groups were examined by two-sample *t*-tests or *χ*^2^ tests. Then, we performed a covariance analysis of composite scores of structural covariance networks controlling for age, gender, education level, and total intracranial volume (TIV) to explore the differences in the structural integrity between the groups.

To test whether the structural covariance networks could predict the MCI development and reversion at the individual level, we established random forest prediction models (Fig. 1B). Random forest algorithm has been proven to have important advantages in terms of robustness to overfitting, ability to handle highly non-linear data, stability in the presence of outliers, and has shown good performance in the processing of neuroimaging data of Alzheimer’s disease [40]. As predictors of random forest, the baseline score maps of the structural covariance network provide information on multiple voxels and the correlation information between voxels, which is an advantage of machine learning in processing high-dimensional and multiple-feature data compared with traditional statistical methods. Fivefold cross-validation and receiver operating characteristic (ROC) analyses were conducted, and the area under the curve (AUC), sensitivity, and specificity were used to evaluate the prediction accuracy. The random forest model was implemented in Python version 3.6.

For random forest classification, measures of the importance of each feature can be calculated based on the reduction in the accuracy of the model when the feature in question (i.e. voxel score) is not included in the subset of features within a tree [41]. We obtained the feature weight distribution based on the random forest model to identify the brain voxels that played a key role in the prediction. The feature weight represents the predicted contribution of a given region to the change in the individual clinical status.

Additionally, we examined whether the GM density of individual brain regions or the atrophy synchronization of brain regions was more sensitive to the early stages of MCI development and reversion. Based on the automated anatomical atlas (http://www.gin.cnrs.fr/AAL), we identified the clusters of voxels that play a key role in the above prediction model as key brain regions. Using the same methodology, we built prediction models and evaluated their prediction accuracy based on the baseline GM density of key brain regions.

We used SPSS version 21 (IBM) to complete the basic statistical analysis and the sklearn package in Python (3.6) for prediction analysis.

## Results

### Clinical characteristics of the participants

Demographic and follow-up information of the participants included in this study are summarized in Table 1 and Additional file 1: Table S1. Longitudinal samples from the BABRI included 65 sNC to build structural covariance networks and 28 sNC, 18 pNC, 20 non-rMCI (containing only the MCI who maintained MCI), and 24 rMCI to conduct predictive analyses. The NC and MCI participants were followed up with a mean duration of 37 and 27 months, respectively.

Another longitudinal sample from the ADNI included 47 sNC to build structural covariance networks and 47 sNC, 54 pNC, 65 non-rMCI, and 73 rMCI to conduct predictive analyses. The NC and MCI participants were followed up with a mean duration of 44 and 49 months, respectively.

Overall, there were no significant differences in age, sex, or education levels between participants with sNC and pNC in both samples, while non-rMCI was relatively older (*p* = 0.030 in BABRI and *p* = 0.099 in ADNI) and more homozygous carriers of ε4 Allele of Apolipoprotein E (APOE4) (*p* = 0.001 in ADNI) than rMCI. For general cognitive function as measured by MMSE at baseline, while sNC participants from BABRI presented better cognition than pNC (*p* < 0.001), the pNC showed quite similar cognition with sNC in ADNI (*p* = 0.213). rMCI in both samples were cognitively better than non-rMCI (*p* = 0.066 in the BABRI and *p* < 0.001 in the ADNI). For vascular risk factors (i.e. hypertension, diabetes, hyperlipidemia, smoking history, and BMI-measured obesity), rMCI was more likely to occur in non-smoking individuals in the ADNI sample (*p* =0.009, Additional file 1: Table S2), and no difference in vascular risk factors was found between pNC and sNC.

### Structural covariance networks

**Fig. 2:**
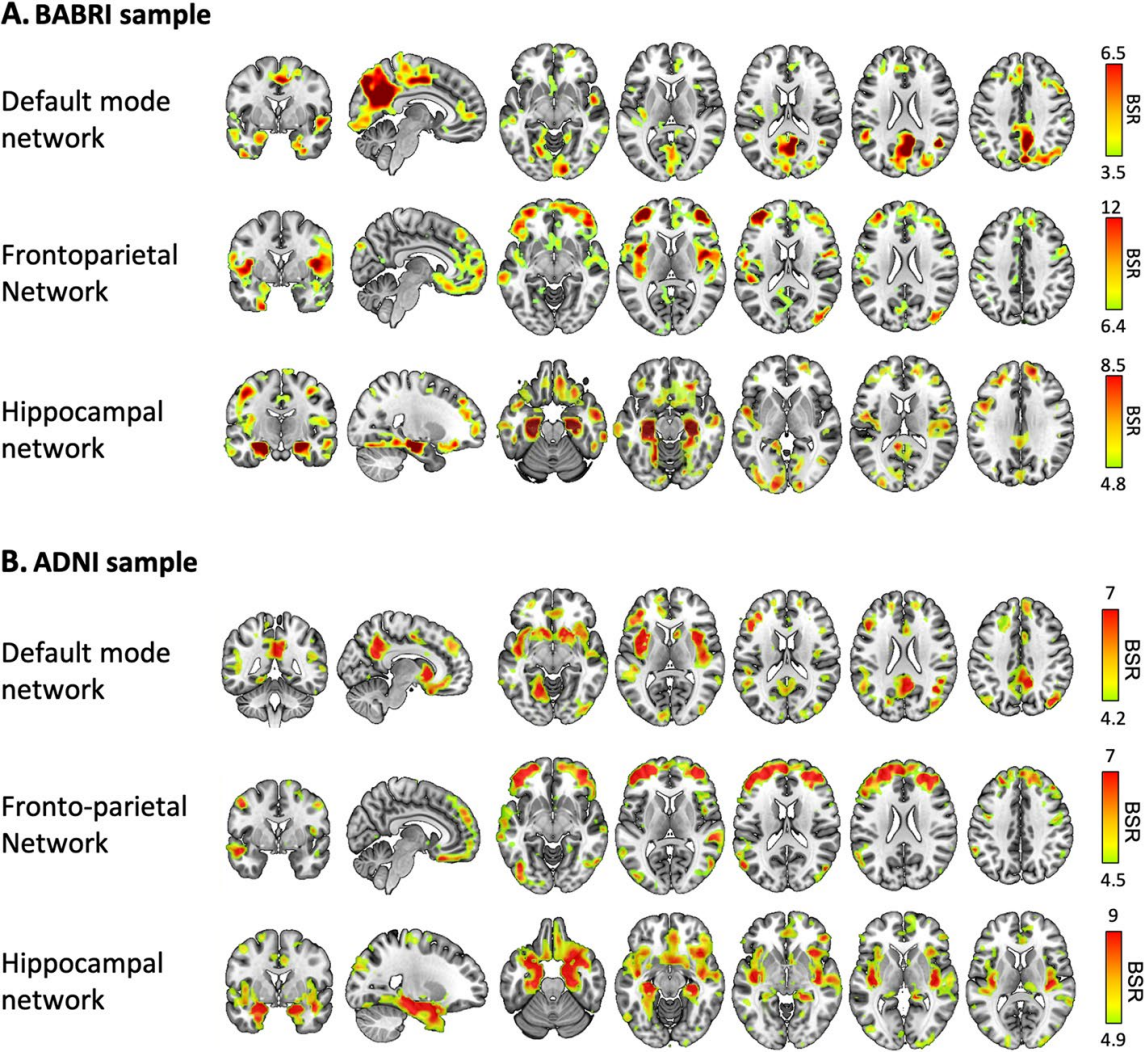
Structural covariance network based on independent cognitively normal elderly from the BABRI. BSR, bootstrap ratio, representing the covariance degree with the seed regions

Seed-PLS analyses were performed on the independent sNC data within the BABRI and ADNI (Fig. 2, Additional file 1: Tables S3-S4). In both the BABRI and ADNI samples, the GM density of the DMN seed regions was mainly covaried with the extended posterior cingulate cortex, superior and middle temporal lobe, middle frontal lobe, and insula. The GM density of the FPN seed regions was mainly covaried with the middle frontal lobe, middle occipital lobe, middle cingulum gyrus, middle temporal lobe, and cuneus. The GM density of the HN seed regions covaried with the extended hippocampal lobe, middle and superior frontal lobe, middle temporal lobe, and other regions.

In the BABRI sample, compared with sNC, pNC had a lower composite score of structural covariance of the FPN (scFN, *p* = 0.030, Table 2) and the HN (scHN, *p* = 0.035), and compared with non-rMCI, rMCI had a higher composite score of scFN (*p* = 0.018) and scHN (*p* = 0.007). The composite score of the structural covariance of the DMN (scDN) also showed a higher trend in rMCI, but it was not significant (*p* = 0.061).

**Table 2:** The differences in structural covariance scores between the groups of the BABRI and ADNI samples

Network	sNC, mean ± SD	pNC, mean ± SD	Non-rMCI, mean ± SD	rMCI, mean ± SD	sNC vs. pNC		Non-rMCI vs. rMCI	
					*F*	*p*	*F*	*p*
BABRI sample								
scDN	1268.2 ± 103.9	1232.8 ± 113.6	1188.3 ± 126.6	1256.6 ± 114.1	2.684	0.109	3.736	0.061
scFN	1679.7 ± 128.2	1614.6 ± 162.2	1562.8 ± 156.3	1660.1 ± 150.5	5.035	0.030	6.101	0.018
scHN	1423.8 ± 117.3	1367.6 ± 125.1	1315.4 ± 129.6	1408.4 ± 130.4	4.741	0.035	8.011	0.007
ADNI sample								
scDN	1595.4 ± 158.9	1476.0 ± 142.1	1491.4 ± 156.2	1569.9 ± 150.0	18.254	< 0.001	20.128	< 0.001
scFN	1323.1 ± 137.5	1239.8 ± 130.3	1253.4 ± 136.2	1300.9 ± 133.3	11.455	0.001	13.309	< 0.001
scHN	1701.4 ± 173.0	1563.9 ± 156.1	1553.5 ± 161.1	1667.9 ± 149.5	18.981	< 0.001	32.399	< 0.001

*Abbreviations*: *sNC* stable normal cognition, *pNC* progressed normal cognition, *Non-rMCI* non-reversed mild cognitive impairment, *rMCI* reversed mild cognitive impairment, *scDN* structural covariance of the default network, *scFN* structural covariance of the frontoparietal control network, *scHN* structural covariance of the hippocampal network

In the ADNI sample, the baseline scores of scDN, scFN, and scHN of sNC and rMCI were higher than those of pNC and non-rMCI (all *p* ≤ 0.001, Table 2). Of note, 34 of 65 (63%) non-rMCI were progressed to AD (pMCI) during the follow-up, and pMCI had lower structural covariation scores than rMCI (Fig. S1, *p* < 0.001) and non-rMCI who maintained MCI (sMCI, *p* < 0.05), adjusted for age, sex, education level, and TIV.

We also examined whether the differences in structural covariation scores were associated with APOE4 carrier status, the strongest known genetic risk factor for late-onset AD cases [42]. We found that APOE4 homozygotes had lower structural covariation scores than non-carriers (Fig. S2, scDN, *p* = 0.017; scFN, *p* = 0.056; scHN, *p* = 0.009) and APOE4 heterozygotes (scDN, *p* = 0.03; scFN, *p* = 0.063; scHN, *p* = 0.025). In addition, the effect of disease duration on MCI reversion was explored in the supplementary analysis (Fig. S3). The longer the disease course of rMCI, the lower the composite score of the structural covariant network (scDN, *r* = − 0.555, *p* = 0.049; scFN, *r* = − 0.513, *p* = 0.073; scHN, *r* = − 0.475, *p* = 0.101).

### Predicting normal-to-MCI progression and MCI-to-normal reversion

The above analyses indicated the possibility to use GM covariance patterns to predict MCI development and reversion several years later at the group level. To investigate whether GM covariance patterns could be useful for predicting MCI development and reversion at the individual level, random forest models were used to construct predictive models for changes in future clinical status.

For N-t-M progression, all structural covariance networks were able to classify cognitively normal elderly people into sNC and pNC based on the BABRI sample (AUC = 0.692–0.792, Table 3). It is worth noting that the hippocampal covariance network achieved the best performance (AUC = 0.792). In addition, based on the ADNI sample, the baseline scores of scDN (AUC = 0.766), scFN (AUC=0.765), and scHN (AUC = 0.785) could also accurately distinguish pNC from sNC (Table 3).

**Table 3:** The prediction results of the development and reversion of MCI based on a structural covariance network

Predictor variable	Normal-to-MCI progression			MCI-to-normal reversion		
	AUC	SEN	SPE	AUC	SEN	SPE
**BABRI sample**						
scDN	0.767 (± 0.109)	0.726	0.747	0.722 (± 0.182)	0.500	0.990
scFN	0.692 (± 0.080)	0.960	0.394	0.745 (± 0.128)	0.440	0.990
scHN	0.792 (± 0.087)	0.860	0.707	0.736 (± 0.151)	0.480	0.990
**ADNI sample**						
scDN	0.766 (± 0.090)	0.774	0.667	0.750 (± 0.089)	0.796	0.636
scFN	0.765 (± 0.096)	0.897	0.556	0.701 (± 0.075)	0.751	0.636
scHN	0.785 (± 0.092)	0.827	0.667	0.809 (± 0.093)	0.679	0.818

*Abbreviations*: *AUC* area under the curve, *SEN* sensitivity, *SPE* specificity, *scDN* structural covariance of the default network, *scFN* structural covariance of the frontoparietal control network, *scHN* structural covariance of the hippocampal network

For M-t-N reversion, the baseline scores of scDN, scFN, and scHN also showed good predictive performance based on the BABRI sample (Table 3, AUC = 0.722–0.745). In the ADNI sample, the baseline scores of scHN achieved the best prediction effect (AUC = 0.809), and the AUCs of the other two prediction models were above 0.701 (Table 3).

**Fig. 3:**
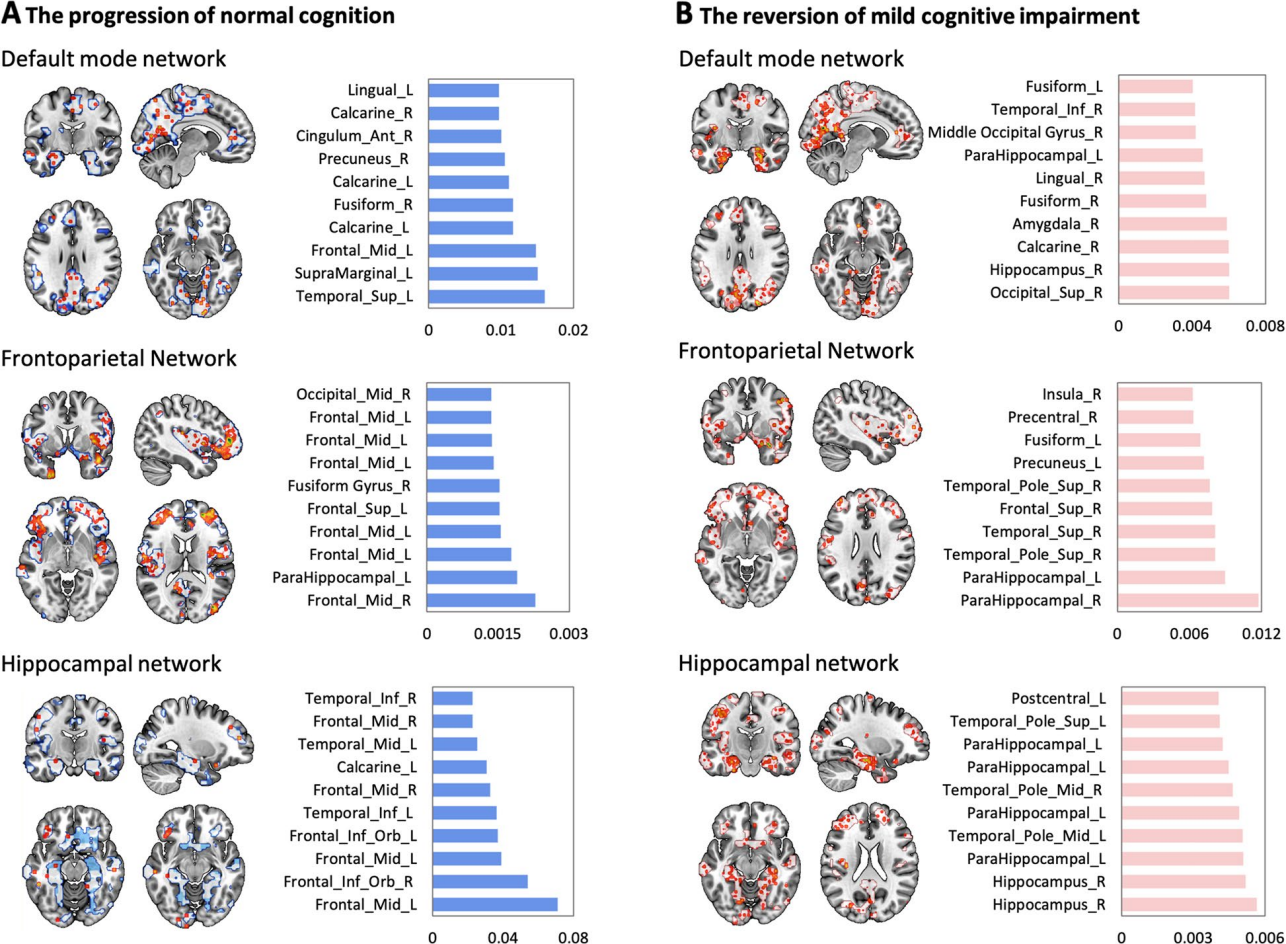
Feature weight distribution of the random forest prediction model of the BABRI sample. The contribution of voxels from the default network, frontoparietal network, and hippocampal network for prediction of the progression of normal cognition (**A**) and the reversion of mild cognitive impairment (**B**). The bar diagram on the right is the cluster with the top 10 feature weights in each network, and the horizontal axis is the weight value. *Abbreviations*: L, left; R, right; Mid, middle; Sup, superior; Ant, anterior; Inf, inferior; Orb, orbital

To further identify the brain regions that play a key role in predicting N-t-M progression and M-t-N reversion, feature weight distributions were depicted in Fig. 3A for the BABRI sample and Fig. S4A for the ADNI sample. We found that for both samples, the superior temporal gyrus of the DMN, and the middle frontal gyrus of the FPN and HN, played a key role in the N-t-M prediction. Notably, the hippocampus and parahippocampal regions played a key role in the M-t-N prediction of all three networks (Fig. 3B, Fig. S4B).

Supplementary analyses were also conducted to clarify the potential impacts of field strength and scanner sites for the ADNI sample, and it turned out that, by including the two factors as covariate variables in the predictive models or only using data with single field strength, all three networks maintained good performance for predictive N-to-M progression and M-to-N reversion (Additional file 1: Tables S6-S9). The prediction of MCI progression and reversion in ADNI samples was also conducted (Additional file 1: Table S5). For MCI progression and reversion, both the structural covariant network and the hippocampus and parahippocampal region showed excellent predictive performance (all AUC > 0.8), especially for scDN (AUC = 0.874) and scHN (AUC = 0.868). More details could be found in the supplementary materials.

### Prediction based on the key brain regions

To explore which of the structural covariance networks and the key brain regions were more sensitive to the development and reversion of MCI, we evaluated the predictive accuracy of key brain regions, that is, regions that emerged as having key roles in the structural covariance networks and hippocampal region (Table 4).

**Table 4:** The prediction results of the development and reversion of MCI based on key brain regions

Normal-to-MCI progression				MCI-to-normal reversion			
Predictor variable	AUC	SEN	SPE	Predictor variable	AUC	SEN	SPE
**BABRI sample**							
Frontal_Mid	0.660 (± 0.111)	0.513	0.798	Hippocampus	0.759 (± 0.160)	0.440	0.990
Temproal_Sup	0.607 (± 0.102)	0.256	0.990	Parahippocampus	0.731 (± 0.182)	0.520	0.990
Hippocampus	0.717 (± 0.129)	0.843	0.566				
**ADNI sample**							
Frontal_Mid	0.655 (± 0.080)	0.465	0.848	Hippocampus	0.780 (± 0.117)	0.530	0.939
Temproal_Sup	0.730 (± 0.128)	0.533	0.848	Parahippocampus	0.769 (± 0.143)	0.676	0.808
Hippocampus	0.803 (± 0.113)	0.587	0.889				

*Abbreviations*: *AUC* area under the curve, *SEN* sensitivity, *SPE* specificity, *scDN* structural covariance of the default network, *scFN* structural covariance of the frontoparietal network, *scHN* structural covariance of the hippocampal network

For N-to-M progression, the predictive accuracy of the middle frontal gyrus, superior temporal gyrus, and hippocampus (AUC = 0.607–0.717) was lower than that of the hippocampus covariance network (AUC = 0.792) based on the BABRI sample, while the predictive accuracy of the hippocampus (AUC = 0.803) was similar to that of the covariance networks based on the ADNI sample.

For M-t-N reversion, the predictive accuracy of individual hippocampal regions (AUC = 0.759) was better than that of the covariance networks based on the BABRI sample, while the predictive accuracy of the hippocampal region (AUC = 0.780) and parahippocampal region (AUC = 0.769) was worse than that of the hippocampus covariance network (AUC = 0.809) based on the ADNI sample.

In addition, we evaluated the GM covariance relationship between these key brain regions. By regressing out the effects of age, sex, education, and TIV, we found that for the N-to-M progression, sNC from BABRI presented a marginally significant positive correlation between the GM density of the superior temporal gyrus and that of the hippocampus (*r* = 0.400, *p* = 0.053), while this correlation disappeared in pNC (*r* = 0.218, *p* = 0.455) in the BABRI sample. Similar results were also identified in the ADNI sample, with sNC (*r* = 0.503, *p* = 0.001), not pNC (*r* = 0.225, *p* = 0.117), having significant GM covariation between the middle frontal gyrus and hippocampus.

And for M-t-N reversion, the GM density of the hippocampal and parahippocampal regions was correlated in both sNC and pNC (*p* < 0.01) in BABRI and ADNI samples.

## Discussion

In the present study, we proposed a prediction framework for the development and reversion of MCI individuals based on the GM structural covariance network. The baseline covariance network scores of DMN, FPN, and HN all predicted N-to-M progression and M-to-N reversion, and HN achieved the optimal prediction performance. These results were replicated in an independent sample from the ADNI.

### The GM covariance network is a good biomarker of MCI development

As the treatment window for AD continues to be advanced, some studies have examined the risk of progression from NC to MCI at the group level [43–45]. However, it is not clear which biomarkers may help predict disease progression at the individual level. An American study was the first to demonstrate on an individual level that cerebrospinal fluid (CSF), MRI, and APOE biomarkers obtained from cognitively normal individuals can be used to predict which individuals will develop clinical symptoms 5, 7, or 10 years after baseline [46]. Similarly, a recent study found that plasma phospho-tau, in combination with brief cognitive tests and APOE genotyping, greatly improves the diagnostic prediction of AD [47].

However, these papers have not focused on the role of interregional synchronization of atrophy (i.e. structural covariation) in the onset of cognitive impairment, either at the group level or at the individual level. In contrast, we found that the hippocampal covariance network score achieved a better predictive outcome (AUC = 0.79, sensitivity = 0.86, specificity = 0.71) in the BABRI sample. This finding has important implications for screening high-risk populations with clinical progression. In the past, we tended to focus on the hippocampus during the development of cognitive impairment [48, 49], but the current study suggests that we also need to consider the synchronization of GM atrophy between the hippocampus and other regions.

Although individual hippocampal regions achieved similar predictive accuracy to the covariance network in ADNI samples, individual brain regions (sensitivity = 0.456–0.587) were less sensitive for predicting MCI development than the covariance network (sensitivity = 0.774–0.897), representing the potential for early identification of individuals at risk of developing MCI.

### The hippocampus is a key area for cognitive improvement in patients with MCI

MCI has often been studied due to its association with dementia, yet higher rates of reversion to normal cognition than progression to dementia suggest that MCI does not necessarily lead to dementia [15, 50]. Although rMCI has received increasing attention in recent years, almost all relevant studies have been limited to describing the rMCI rate and influencing factors, such as higher levels of complex mental activity (e.g. reading books), better vision/smelling ability, and lower diastolic blood pressure [20]. However, these works are far from sufficient to understand the pathological mechanism and to achieve early detection of rMCI. Our study is the first to establish models for predicting future cognitive improvement in MCI at the individual level and the first to explore rMCI at the network scale.

A longitudinal study of MCI reversion to cognitively normal status showed that MCI reverters exhibited less severely decreased functional connectivity in the DMN and executive control networks than non-reverters [51]. From the perspective of structural networks, the current research also shows that the FPN and HN of rMCI have better structural integrity than non-rMCI (Table 2). Other evidence suggests that cognitive improvement in MCI is associated with greater cortical thickness in the right parahippocampal gyrus and greater density in the left hippocampus at baseline [21, 51]. These findings support the results of the current study: the hippocampal region plays a leading role in the early prediction of MCI reversion. Therefore, improvement of the function of the hippocampal region is the key to intervention training and early treatment of MCI [52].

### Brain mechanisms of MCI development and reversion

**Fig. 4:**
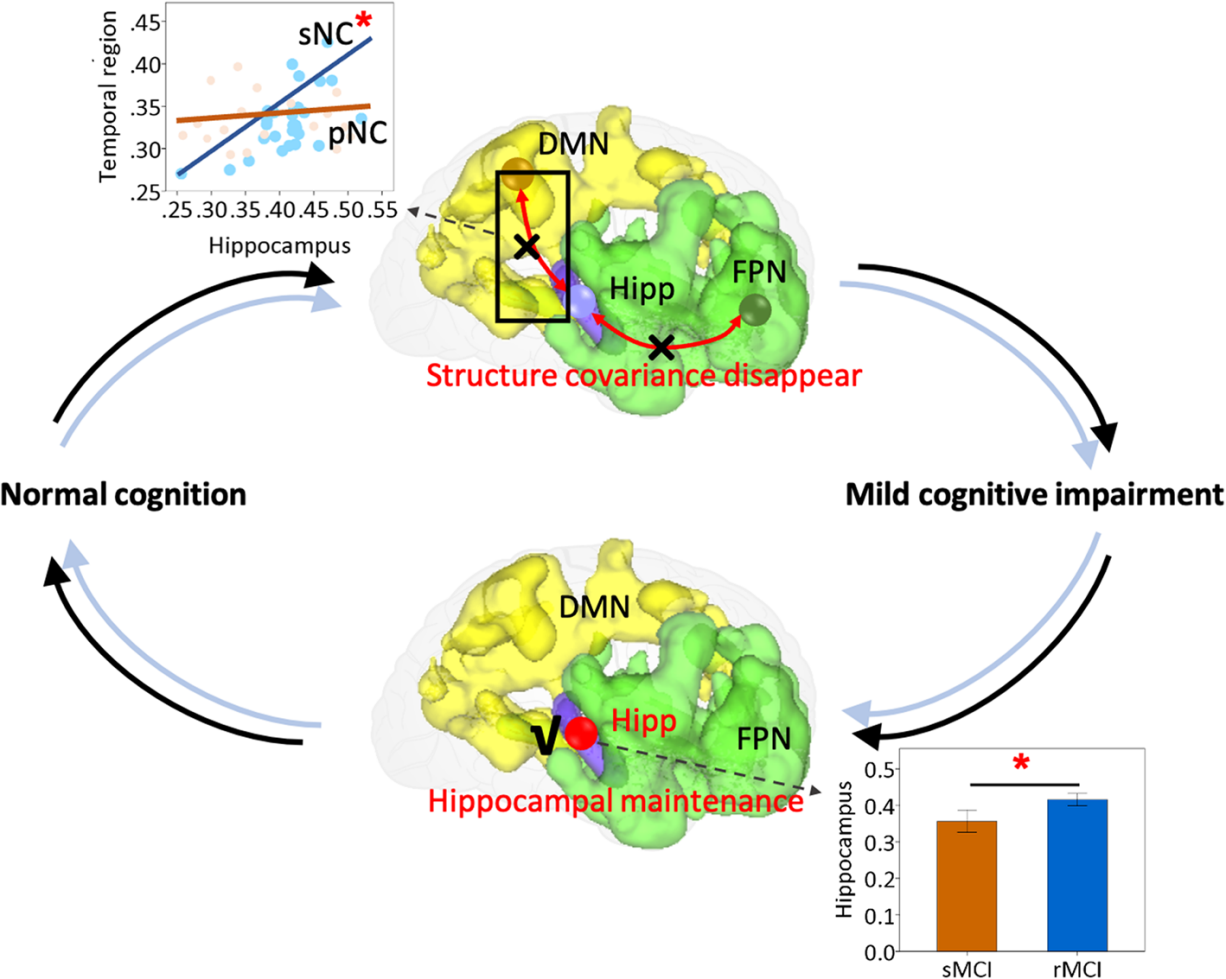
Possible brain mechanisms underlying the development and reversion of mild cognitive impairment. For the development of mild cognitive impairment, the disappearance of atrophy synchronism between the hippocampal region and the frontal and lateral temporal lobes played a key role, while the reversion of mild cognitive impairment was mainly due to accelerated atrophy in the hippocampus. *Abbreviations*: DMN, default mode network; FPN, frontoparietal network; Hipp, hippocampal; sNC, stable normal cognition; pNC, progressive normal cognition; non-rMCI, non-reversed mild cognitive impairment; rMCI, reversed mild cognitive impairment

The N-to-M progression in normal older adults and M-to-N reversion in MCI patients seem to involve different brain mechanisms (Fig. 4). For N-to-M progression, the predictive performance of the structural covariance network is better than that of the GM density of any single brain region in the BABRI sample, which may be due to the correlation between multiple features (brain regions) providing additional predictive information. In other words, the synchronous loss of GM atrophy in the hippocampus and other regions (such as the frontal and lateral temporal lobes) may precede the atrophy of individual regions (e.g. hippocampus, medial temporal lobe) before MCI development (that is, N-to-M progression). However, when cognitive impairment has already occurred, the hippocampus plays a leading role in influencing cognition, as both the GM covariance network score of HM and the GM density of the hippocampus are good predictors of M-t-N reversion.

### Factors associated with reversion from MCI to NC status

Current studies showed that non-APOE4 homozygotes and non-smokers are more likely to reverse the MCI to the NC state. For APOE4 carrier status and GM covariance network, we found that the GM structural integrity of APOE4 homozygous, not APOE4 heterozygous, was worse than that of non-carriers (Fig. S2). Similarly, previous studies show accelerated cognitive decline in APOE4 homozygotes but not heterozygotes [53–55]. Furthermore, the association between APOE4 and cognition and AD risk is thought to be modified by age and family history of dementia. A recent 20-year follow-up study supported a complex antagonist pleiotropic effect of APOE4 heterozygosity over the adult life course, characterized by cognitive advantage in midlife [56]. Another study showed an increased risk of early-onset AD in APOE4 heterozygotes only in participants with a positive family history [57]. Future studies need to further explore the association between APOE4 carrier status and the structural integrity of GM, considering the age and family history of the participants.

In addition, we found that rMCI was more likely to occur in non-smoking individuals in the ADNI sample (Additional file 1: Table S3). Two other recent studies have reached the same conclusion that non-smoking is a beneficial factor in reversing from MCI to NC state [58, 59]. However, another study found no differences in vascular risk factors between the MCI reversion, stabilization, and progression groups, with low white matter hyperintensity grades characteristic of MCI reversion [60]. Taken altogether, the findings on cerebrovascular factors related to MCI development and reversion are few, and no consistent conclusions have been reached.

## Limitations

This study has several limitations. First, the observed sample size of the current study was small, although the reliability of the study results was confirmed by a cross-validation strategy and the use of independent samples. Second, this study only focused on the measurement of structural covariance and lacked a comparison or combination with other biomarkers that have been proven to be related to the development or reversion of MCI, such as CSF amyloid-β 1 to 42 peptide and CSF total tau [11, 21, 46]. Finally, only baseline data were used in the current study, and the predictive performance could be boosted if longitudinal data were incorporated into the model. These questions that have not been verified in the current study need to be further explored in future studies.

## Conclusion

The present study first demonstrated, at the individual level, that structural covariance networks could serve as biomarkers for MCI development and reversion. The baseline scores of GM covariance among DMN, FPN, and HN accurately predicted normal-to-MCI progression (AUC = 0.692–0.792) and MCI-to-normal reversion (AUC=0.701-0.809). In addition, the synchronous loss of GM atrophy in the hippocampus and other regions may be more sensitive to MCI development and reversion than GM atrophy in individual brain regions. These findings provide more evidence about the mechanism underlying MCI development and new neuroimaging targets for the early screening of individuals at high risk of developing MCI.

## Supplementary Information

**Additional file 1: Table S1.** The follow-up information of the NC subjects included in this study. **Table S2.** Vascular risk factors of the BABRI and ADNI samples. **Table S3.** List of regions showing covariance between the seed network regions and whole brain patterns of the grey matter of BABRI. **Table S4.** List of regions showing covariance between the seed network regions and whole brain patterns of the grey matter of ADNI. **Table S5.** The differences in structural covariance scores between groups of the ADNI samples, adjusted for age, sex, education level, TIV, field strength, and scan site. **Table S6.** The prediction results of the development and reversion of MCI of the ADNI samples, adjusted for field strength and scan site. **Table S7.** The differences in structural covariance scores between groups of the 1.5T field intensity in the ADNI samples, adjusted for age, sex, education level, and TIV. **Table S8.** The prediction results of the development and reversion of MCI of the 1.5T field intensity in the ADNI samples. **Table S9.** Prediction of MCI progression, stabilization, and reversion in ADNI samples. **Fig. S1.** Differences in structural covariance scores of rMCI, sMCI, and pMCI of the ADNI sample. **Fig. S2.** The relationship between the composite score of the structural covariance network and the APOE4 carrier status of the ADNI sample. **Fig. S3.** Effect of disease course on MCI reversion of the ADNI sample. **Fig. S4.** Feature weight distribution of the random forest prediction model of the ADNI sample. **Fig. S5.** The relationship between the composite score of the structural covariance network and the grey matter density of the hippocampus.

## Data Availability

The datasets used and/or analysed during the current study are available from the corresponding author upon reasonable request.

## Abbreviations

MCI: Mild cognitive impairment
AD: Alzheimer’s disease
N-t-M: Progression from normal cognition to MCI
M-t-N: Reversion from MCI to normal cognition
NC: Normal cognition
GM: Grey matter
MMSE: Mini-Mental State Examination
seed-PLS: Seed-based partial least squares
DMN: Default network
FPN: Frontoparietal network
scDN: Structural covariance of the default network
scFN: Structural covariance of the frontoparietal control network
scHN: Structural covariance of the hippocampal network
