# Supplementary material for: Hippocampus-centred grey matter covariance networks predict the development and reversion of mild cognitive impairment
Source: Alzheimers Res Ther. 2023 Feb 2;15:27. doi: 10.1186/s13195-023-01167-z (PMC9893696; doi:10.1186/s13195-023-01167-z)
Supplement: Supplementary file 1 — Additional file 1: Table S1. The follow-up information of the NC subjects included in this study. Table S2. Vascular risk factors of the BABRI and ADNI samples. Table S3. List of regions showing covariance between the seed network regions and whole brain patterns of the grey matter of BABRI. Table S4. List of regions showing covariance between the seed network regions and whole brain patterns of the grey matter of ADNI. Table S5. The differences in structural covariance scores between groups of the ADNI samples, adjusted for age, sex, education level, TIV, field strength, and scan site. Table S6. The prediction results of the development and reversion of MCI of the ADNI samples, adjusted for field strength and scan site. Table S7. The differences in structural covariance scores between groups of the 1.5T field intensity in the ADNI samples, adjusted for age, sex, education level, and TIV. Table S8. The prediction results of the development and reversion of MCI of the 1.5T field intensity in the ADNI samples. Table S9. Prediction of MCI progression, stabilization, and reversion in ADNI samples. Fig. S1. Differences in structural covariance scores of rMCI, sMCI, and pMCI of the ADNI sample. Fig. S2. The relationship between the composite score of the structural covariance network and the APOE4 carrier status of the ADNI sample. Fig. S3. Effect of disease course on MCI reversion of the ADNI sample. Fig. S4. Feature weight distribution of the random forest prediction model of the ADNI sample. Fig. S5. The relationship between the composite score of the structural covariance network and the grey matter density of the hippocampus. [file 13195_2023_1167_MOESM1_ESM.docx]

**Supplementary** **materials**

**Follow-up information**

Most of the ADNI subjects were followed every 6 months, and the BABRI subjects were followed every 12 or 18 months. The follow-up information of the subjects is summarized in Table S1. Compared with ADNI subjects, BABRI subjects had a shorter follow-up time, especially MCI subjects.

**The effect of** **disease duration on MCI reversion**

Not all subjects have reported their duration of cognitive impairment at baseline or recruitment. 20% of MCI participants in the ADNI sample had baseline disease duration (rMCI=17, non-rMCI=10). The results showed that rMCI (Fig. S3, mean duration=3.5 years) had a shorter duration at baseline than non-rMCI (mean duration=4.9 years), but there was no statistical difference (p>0.05). As a new finding, the longer the disease course of rMCI, the lower the composite score of the structural covariant network (scDN, r=-0.555, p=0.049; scFN, r=-0.513, p=0.073; scHN, r=-0.475, p=0.101), corrected for demographic information and TIV. To some extent, this finding indicated that the longer the time of MCI, the worse the structural integrity of gray matter, and the more difficult to revert to the NC state.

**Effects of field strength and scan sites**

As the imaging data of the BABRI sample were acquired under identical field strengths, the possible effects of different field strengths were mainly in the ADNI sample. Several supplementary analyses were performed to clarify the influence of field strength on our results, on the one hand, we repeated all the analyses using the field strength as the covariable (Table S6-S7), and on the other hand, we only included MRI data with a field intensity of 1.5T and repeated all analyses (Table S8-S9). Due to the small number of non-rMCI participants (n=4) with 3T field intensity in the ADNI sample, MRI data with 3T cannot be used for repeatability verification.

**Result 1.** After adjusting for demographic information, field strength, and scanner sites, the baseline scores of scDN, scFN, and scHN of sNC were higher than those of pNC (all p≤0.001, Table S6). Compared with non-rMCI, rMCI had a higher composite score of scHN (p=0.041). The composite score of scFN (p=0.053) and scDN (p=0.094) also showed a higher trend in rMCI but it was not significant.

At the individual level, the prediction results of the random forest model show a consistent trend with the main results in the manuscript. For normal-to-MCI progression, all structural covariance networks were able to classify cognitively normal elderly into sNC and pNC (AUC=0.725-0.785, Table S7), and the predictive accuracy of the middle frontal gyrus (AUC=0.638) and superior temporal gyrus (AUC=0.738) was lower than that of the scDN and scHN, while the predictive accuracy of the hippocampus (AUC=0.802) was similar to that of the covariance networks. For MCI-to-normal reversion, the predictive accuracy of individual hippocampal (AUC=0.826) or parahippocampus (AUC=0.834) regions was better than that of the covariance networks (AUC=0.796-0.811).

**Result 2.** In the ADNI participants with 1.5T MRI, the baseline scores of scDN, scFN, and scHN of sNC and rMCI were higher than those of pNC (all p≤0.002, Table S8) and non-rMCI (all p≤0.014). The prediction results of the random forest model show a consistent trend with *result 1* (Table S9).

**Prediction of MCI progression, stabilization, and reversion in ADNI samples**

We further compared the GM covariance characteristics of rMCI with sMCI (MCI who maintained MCI) and pMCI (MCI who progressed to AD). For MCI maintenance and reversion, individual hippocampal regions (AUC=0.748, Table S5) had better predictive accuracy than covariance networks (AUC=0.688-0.731), which is consistent with the results in the BABRI sample. For MCI progression and reversion, both the structural covariant network and the hippocampus and parahippocampal region showed excellent predictive performance (all AUC>0.8), especially for scDN (AUC=0.874) and scHN (AUC= 0.868).

**Table S1.** **The follow-up information of the NC and MCI subjects included in this study.**

| **Cohorts** | **Number of subjects** | **Follow-up months**  **(1^st^, 3^rd^)** | **Progress/ Revert (Yes/No)** | **Termination status (n)** | **Months to reversion/**  **maintain/progression**  **(1^st^, 3^rd^)** |
| --- | --- | --- | --- | --- | --- |
| **NC subjects with follow-up** | | | | | |
| BABRI | 46 | 37 (19, 53) | No | NC (28) | Months to maintain:  35 (21, 42) |
|  |  |  | Yes | MCI (18) | Months to progression:  29 (13, 54) |
| ADNI | 101 | 44 (24, 60) | No | NC (47) | Months to maintain:  35 (24, 48) |
|  |  |  | Yes | MCI (54) | Months to progression:  30 (24, 36) |
| **MCI subjects with follow-up** | | | | | |
| BABRI | 44 | 27 (17, 36) | Yes | NC (24) | Months to reversion:  12 (5, 16) |
|  |  |  | No | MCI (20) | Months to maintain:  23 (16, 31) |
| ADNI | 138 | 49 (36, 60) | Yes | NC (73) | Months to reversion:  31 (12, 48) |
|  |  |  | No | MCI/AD (65) | Months to maintain/progression:  35 (24, 42) |

Abbreviations: n, Number of subjects

**Table S2. Vascular risk factors of the BABRI and ADNI samples.**

|  | **sNC** | **pNC** | ***T/χ^2^*** | ***p*** | **non-rMCI** | **rMCI** | ***T/χ^2^*** | ***p*** |
| --- | --- | --- | --- | --- | --- | --- | --- | --- |
| **BABRI sample** | | | | | | | | |
| No. Participants | 28 | 18 | — | — | 20 | 24 | — | — |
| Hypertension, % | 7 (25%) | 5 (27.8%) | 0.105 | 0.746 | 5 (25%) | 12 (50%) | 2.876 | 0.090 |
| Diabetes, % | 5 (17.9%) | 1 (5.9%) | 1.313 | 0.252 | 2 (10%) | 2 (8.3%) | 0.037 | 0.848 |
| Hyperlipidemia, % | 10 (43.5%) | 6 (37.5%) | 0.139 | 0.709 | 3 (27.3%) | 8 (38.1%) | 0.375 | 0.540 |
| Smoking, % | 8 (29.6%) | 3 (17.6%) | 0.799 | 0.371 | 3 (18.8%) | 7 (33.3%) | 0.979 | 0.322 |
| BMI | 25.8±2.9 | 24.2±2.9 | 1.829 | 0.074 | 23.9±4.0 | 25.1±2.7 | -1.185 | 0.243 |
| **ADNI sample** | | | | | | | | |
| No. Participants | 47 | 54 | — | — | 65 | 73 | — | — |
| Hypertension, % | 18 (38.3%) | 23 (42.6%) | 0.192 | 0.661 | 24 (36.9%) | 26 (35.6%) | 0.025 | 0.873 |
| Diabetes, % | 2 (4.3%) | 5 (9.3%) | 0.975 | 0.323 | 5 (7.7%) | 3 (4.1%) | 0.808 | 0.369 |
| Hyperlipidemia, % | 18 (38.3%) | 24 (44.4%) | 0.391 | 0.532 | 24 (36.9%) | 24 (32.9%) | 0.248 | 0.618 |
| Smoking, % | 8 (17%) | 9 (16.7%) | 0.002 | 0.962 | 16 (24.6%) | 6 (8.2%) | 6.898 | **0.009** |
| BMI | 26.2±4.0 | 27.1±5.1 | -0.962 | 0.338 | 26.0±4.1 | 27.2±5.5 | -1.592 | 0.062 |
| SBP | 137.4±16.7 | 135.6±18.5 | 0.521 | 0.604 | 134.9±16.6 | 131.7±14.6 | 1.219 | 0.225 |
| DBP | 76.2±9.3 | 74.9±9.2 | 0.705 | 0.483 | 75.0±9.9 | 73.9±8.8 | 0.712 | 0.478 |

Abbreviations: BMI, body mass index; SBP, systolic blood pressure; DBP, diastolic blood pressure.

**Table S3. List of regions showing covariance between the seed network regions and whole brain patterns of grey matter of BABRI.**

| **Lat** | **Peak MNI coordinate region** | **Number of voxels** | **Peak MNI coordinate** | | | **Peak BSR** |
| --- | --- | --- | --- | --- | --- | --- |
|  |  |  | **x** | **y** | **z** |  |
| **Default mode network** | | | | | | |
| L | Cingulum_Post | 5549 | 0 | -48 | 27 | 12.86 |
| L | SupraMarginal | 217 | -48 | -45 | 27 | 6.74 |
| R | Temporal_Sup | 176 | 51 | 0 | -6 | 6.56 |
| R | Temporal_Mid | 158 | 63 | -42 | 0 | 6.34 |
| R | Frontal_Mid | 153 | 30 | 21 | 39 | 6.15 |
| L | Insula | 263 | -42 | -21 | -3 | 5.45 |
| L | Temporal_Inf | 449 | -54 | -42 | -18 | 5.13 |
| **Frontoparietal network** | | | | | | |
| L | Frontal_Mid | 6886 | -33 | 54 | 12 | 27.62 |
| R | Occipital_Mid | 173 | 45 | -81 | 18 | 11.34 |
| L | Cingulum_Mid | 121 | -9 | -36 | 39 | 10.95 |
| L | Temporal_Mid | 146 | -69 | -30 | -9 | 9.97 |
| L | Cuneus | 340 | -12 | -48 | 18 | 9.46 |
| **Hippocampal network** | | | | | | |
| R | Hippocampus | 6044 | 27 | -21 | -15 | 46.64 |
| L | Frontal_Mid | 469 | -27 | 39 | 27 | 10.60 |
| R | Frontal_Sup | 487 | 15 | 51 | 33 | 9.04 |
| R | Temporal_Mid | 145 | 42 | -66 | 6 | 8.71 |
| R | Paracentral_Lobule | 133 | 9 | -24 | 72 | 7.87 |
| R | Temporal_Inf | 107 | 69 | -33 | -24 | 7.48 |
| L | Temporal_Pole_Sup | 266 | -42 | 9 | -24 | 7.32 |

The table show clusters with cluster size (number of voxels) more than 100 in three covariant networks. Abbreviations: BSR, bootstrap ratio equivalent to a z-score; Post, posterior; Sup, superior; Mid, middle; Inf, inferior; Med, medial; L, left; R, right.

**Table S4. List of regions showing covariance between the seed network regions and whole brain patterns of grey matter of ADNI.**

| **Lat** | **Peak MNI coordinate region** | **Number of voxels** | **Peak MNI coordinate** | | | **Peak BSR** |
| --- | --- | --- | --- | --- | --- | --- |
|  |  |  | **x** | **y** | **z** |  |
| **Default mode network** | | | | | | |
| L | Cingulate_Post | 4460 | 0 | -48 | 27 | 44.73 |
| R | Frontal_Med_Orb | 2261 | 15 | 54 | -3 | 17.15 |
| R | Occipital_Mid | 704 | 36 | -75 | 27 | 14.16 |
| L | Frontal_Mid_2 | 278 | -30 | 42 | 24 | 11.05 |
| **Frontoparietal network** | | | | | | |
| L | Frontal_Mid_2 | 5465 | -36 | 51 | 9 | 42.98 |
| R | Insula | 721 | 45 | 0 | 6 | 16.44 |
| L | Calcarine | 937 | -3 | -96 | 9 | 15.73 |
| L | Temporal_Inf | 279 | -51 | -45 | -15 | 15.35 |
| L | Occipital_Mid | 162 | -33 | -93 | 0 | 13.67 |
| L | Cingulate_Mid | 131 | -6 | -33 | 42 | 11.91 |
| **Hippocampal network** | | | | | | |
| R | ParaHippocampal | 6381 | 27 | -21 | -18 | 44.95 |
| L | Frontal_Mid_2 | 458 | -27 | 39 | 27 | 12.79 |
| R | Frontal_Sup_2 | 490 | 18 | 57 | -3 | 11.43 |
| R | Temporal_Mid | 153 | 45 | -69 | 9 | 8.63 |
| R | Precentral | 242 | 48 | 9 | 33 | 8.52 |

The table show clusters with cluster size (number of voxels) more than 100 in three covariant networks. Abbreviations: BSR, bootstrap ratio equivalent to a z-score; Post, posterior; Med, medial; Orb, orbital; Mid, middle; Inf, inferior; Sup, superior; L, left; R, right.

**Table S5. Prediction of MCI progression, stabilization, and reversion in ADNI samples.**

| **sMCI vs. rMCI** | | | | **pMCI vs. rMCI** | | | |
| --- | --- | --- | --- | --- | --- | --- | --- |
| **Predictor variable** | **AUC** | **SEN** | **SPE** | **Predictor variable** | **AUC** | **SEN** | **SPE** |
| **Structural covariance network** | | | | | | | |
| scDN | 0.714 (+/- 0.155) | 0.631 | 0.798 | scDN | 0.874 (+/- 0.042) | 0.675 | 0.919 |
| scFN | 0.688 (+/- 0.083) | 0.756 | 0.596 | scFN | 0.802 (+/- 0.073) | 0.908 | 0.566 |
| scHN | 0.731 (+/- 0.118) | 0.489 | 0.909 | scHN | 0.868 (+/- 0.091) | 0.7 | 0.919 |
| **key brain regions** | | | | | | | |
| Hippocampus | 0.748 (+/- 0.138) | 0.536 | 0.909 | Hippocampus | 0.842 (+/- 0.105) | 0.665 | 0.919 |
| Parahippocampus | 0.711 (+/- 0.118) | 0.542 | 0.909 | Parahippocampus | 0.832 (+/- 0.072) | 0.725 | 0.838 |

Abbreviations: sMCI, stable mild cognitive impairment; rMCI, reversed mild cognitive impairment; pMCI, progressive mild cognitive impairment; AUC, area under the curve; SEN, sensitivity; SPE, specificity; scDN, structural covariance of the default network; scFN, structural covariance of the frontoparietal network; scHN, structural covariance of the hippocampal network.

**Table S6.** **The differences in structural covariance scores between groups of the ADNI samples, adjusted for age, sex, education level, TIV, field strength, and scan site.**

| **Network** | **sNC *vs.* pNC** | | **non-rMCI *vs.* rMCI** | |
| --- | --- | --- | --- | --- |
|  | ***F*** | ***p*** | ***F*** | ***p*** |
| **scDN** | 18.005 | **<0.001** | 2.846 | 0.094 |
| **scFN** | 11.437 | **0.001** | 3.817 | 0.053 |
| **scHN** | 18.226 | **<0.001** | 4.252 | **0.041** |

Abbreviations: sNC, stable normal cognition; pNC, progressive normal cognition; non-rMCI, non-reversed mild cognitive impairment; rMCI, reversed mild cognitive impairment; scDN, structural covariance of the default network; scFN, structural covariance of the frontoparietal control network; scHN, structural covariance of the hippocampal network.

**Table S7. The prediction results of the development and reversion of MCI of the ADNI samples, adjusted for field strength and scan site.**

| **Normal-to-MCI progression** | | | | **MCI-to-normal reversion** | | | |
| --- | --- | --- | --- | --- | --- | --- | --- |
| **Predictor variable** | **AUC** | **SEN** | **SPE** | **Predictor variable** | **AUC** | **SEN** | **SPE** |
| **Structural covariance network** | | | | | | | |
| scDN | 0.772 (+/- 0.066) | 0.726 | 0.707 | scDN | 0.809 (+/- 0.088) | 0.851 | 0.616 |
| scFN | 0.725 (+/- 0.074) | 0.776 | 0.667 | scFN | 0.796 (+/- 0.043) | 0.781 | 0.758 |
| scHN | 0.785 (+/- 0.102) | 0.593 | 0.848 | scHN | 0.811 (+/- 0.090) | 0.599 | 0.919 |
| **key brain regions** | | | | | | | |
| Frontal_Mid | 0.638 (+/- 0.125) | 0.405 | 0.859 | Hippocampus | 0.826 (+/- 0.119) | 0.673 | 0.909 |
| Temproal_Sup | 0.738 (+/- 0.132) | 0.834 | 0.556 | Parahippocampus | 0.834 (+/- 0.097) | 0.789 | 0.747 |
| Hippocampus | 0.802 (+/- 0.104) | 0.576 | 0.889 |  |  |  |  |

Abbreviations: AUC, area under the curve; SEN, sensitivity; SPE, specificity; scDN, structural covariance of the default network; scFN, structural covariance of the frontoparietal network; scHN, structural covariance of the hippocampal network.

**Table S8. The differences in structural covariance scores between groups of the 1.5T field intensity in the ADNI samples, adjusted for age, sex, education level, and TIV.**

| **Network** | **sNC *vs.* pNC** | | **non-rMCI *vs.* rMCI** | |
| --- | --- | --- | --- | --- |
|  | ***F*** | ***p*** | ***F*** | ***p*** |
| scDN | 16.385 | **<0.001** | 11.844 | **0.001** |
| scFN | 10.419 | **0.002** | 6.269 | **0.014** |
| scHN | 17.88 | **<0.001** | 17.699 | **<0.001** |

Abbreviations: sNC, stable normal cognition; pNC, progressive normal cognition; non-rMCI, non-reversed mild cognitive impairment; rMCI, reversed mild cognitive impairment; scDN, structural covariance of the default network; scFN, structural covariance of the frontoparietal control network; scHN, structural covariance of the hippocampal network.

**Table S9. The prediction results of the development and reversion of MCI of the 1.5T field intensity in the ADNI samples**

| **Normal-to-MCI progression** | | | | **MCI-to-normal reversion** | | | |
| --- | --- | --- | --- | --- | --- | --- | --- |
| **Predictor variable** | **AUC** | **SEN** | **SPE** | **Predictor variable** | **AUC** | **SEN** | **SPE** |
| **Structural covariance network** | | | | | | | |
| scDN | 0.702 (+/- 0.240) | 0.519 | 0.990 | scDN | 0.701 (+/- 0.133) | 0.429 | 0.990 |
| scFN | 0.689 (+/- 0.202) | 0.462 | 0.990 | scFN | 0.598 (+/- 0.196) | 0.229 | 0.990 |
| scHN | 0.685 (+/- 0.156) | 0.394 | 0.990 | scHN | 0.757 (+/- 0.077) | 0.468 | 0.990 |
| **key brain regions** | | | | | | | |
| Frontal_Mid | 0.586 (+/- 0.135) | 0.431 | 0.747 | Hippocampus | 0.761 (+/- 0.126) | 0.531 | 0.990 |
| Temproal_Sup | 0.688 (+/- 0.068) | 0.843 | 0.495 | Parahippocampus | 0.740 (+/- 0.127) | 0.540 | 0.990 |
| Hippocampus | 0.731 (+/- 0.092) | 0.518 | 0.990 |  |  |  |  |

Abbreviations: AUC, area under the curve; SEN, sensitivity; SPE, specificity; scDN, structural covariance of the default network; scFN, structural covariance of the frontoparietal network; scHN, structural covariance of the hippocampal network.

**
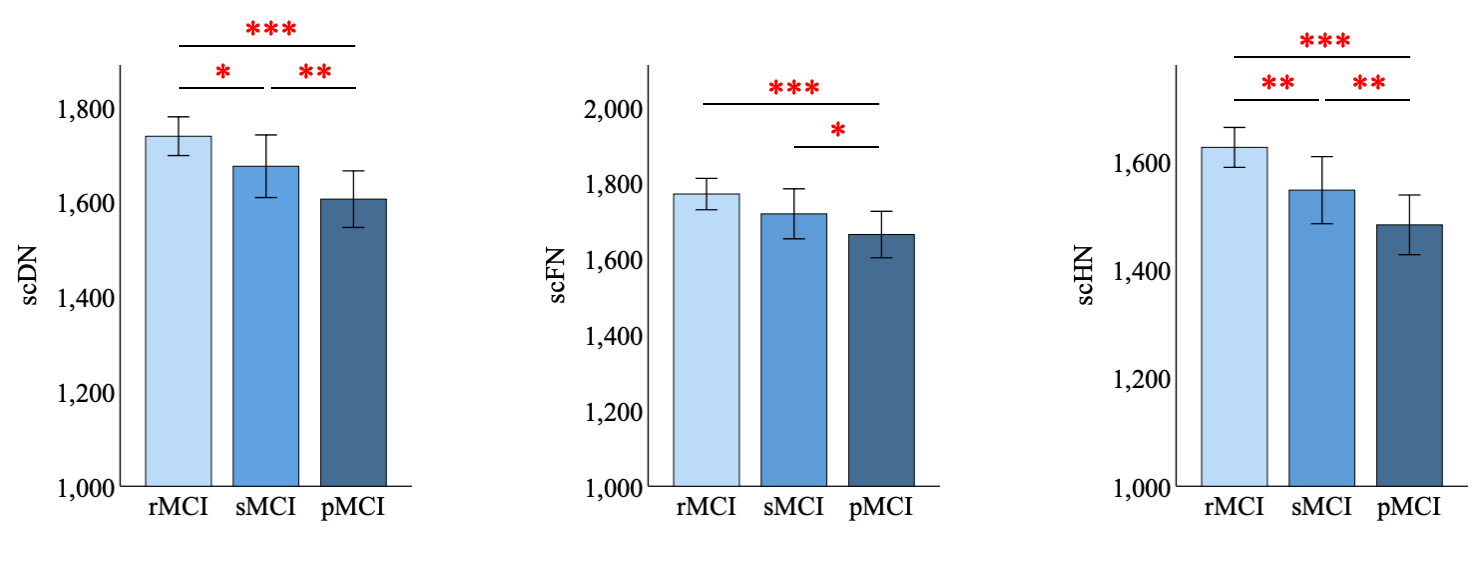
**

**Fig. S1 Differences in structural covariance scores of rMCI, sMCI and pMCI of the ADNI sample.** Abbreviations: scDN, structural covariance of the default network; scFN, structural covariance of the frontoparietal control network; scHN, structural covariance of the hippocampal network; rMCI, MCI that reverted to normal cognition; sMCI, MCI that maintain to MCI; pMCI, MCI that progressed to AD; *p<0.05, **p<0.01, ***p<0.001.

**
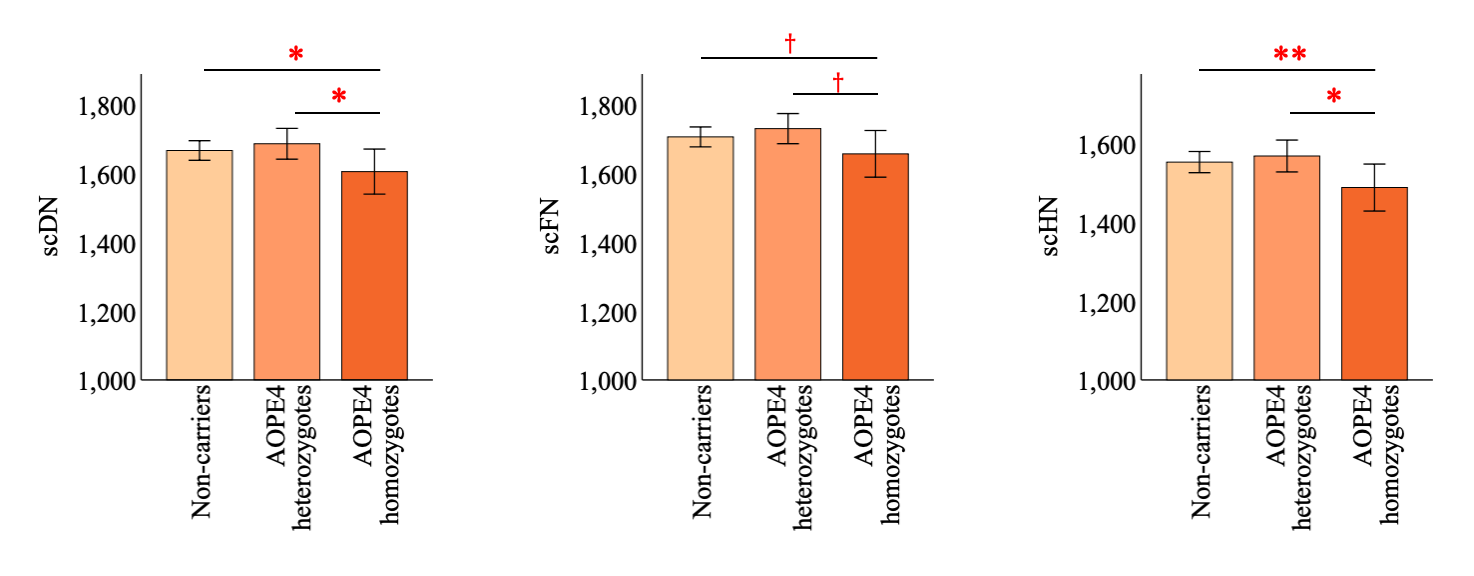
**

**Fig. S2 The relationship between the composite score of the structural covariance network and** **the APOE4 carrier status of the ADNI sample.** Abbreviations: scDN, structural covariance of the default network; scFN, structural covariance of the frontoparietal control network; scHN, structural covariance of the hippocampal network; ^†^p<0.07, *p<0.05, **p<0.01, ***p<0.001.


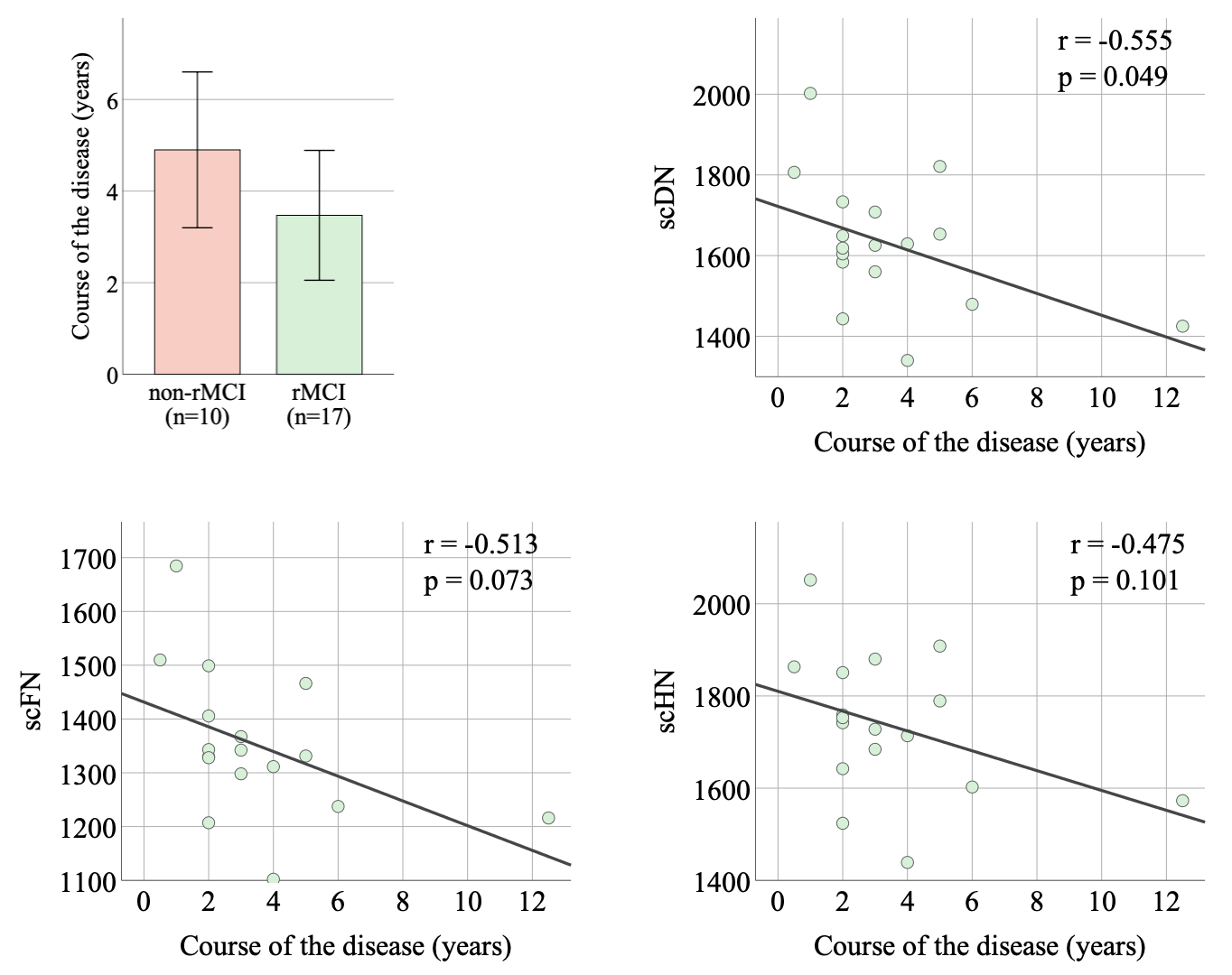


**Fig. S3 Effect of disease course on MCI reversion of the ADNI sample.** After adjusting for age, sex, education, and TIV, the results of the two-sample t-test showed no significant difference in the course of disease between rMCI and non-rMCI at baseline. After adjusting for age, sex, education, and TIV, the results of correlation analysis showed that the combined scores of scDN and scFN were marginally correlated with the course of the disease.

**
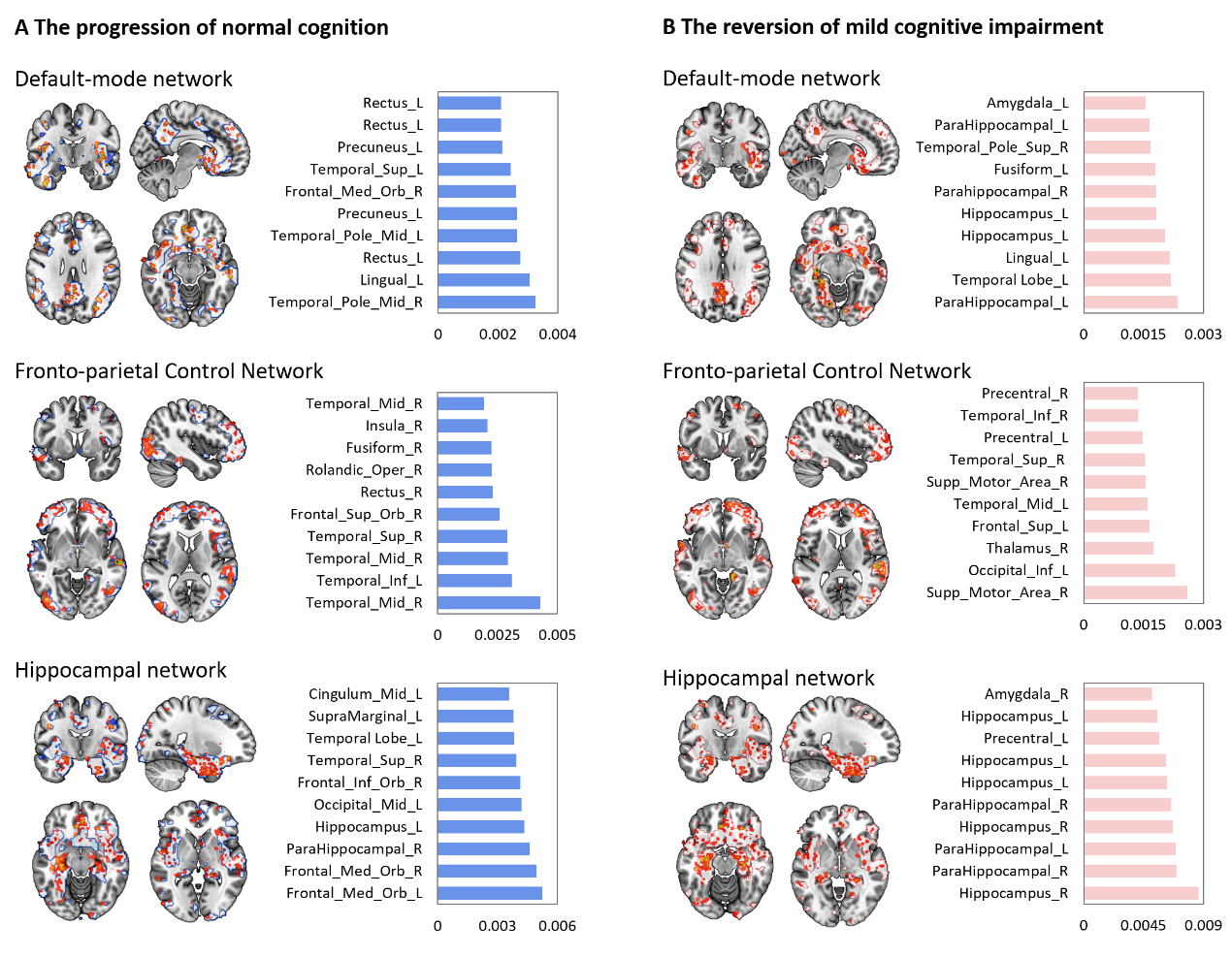
**

**Fig. S4 Structural covariance networks based on independent cognitive normal elderly of ADNI.** BSR: bootstrap ratio, representing the covariance degree with the seed regions.


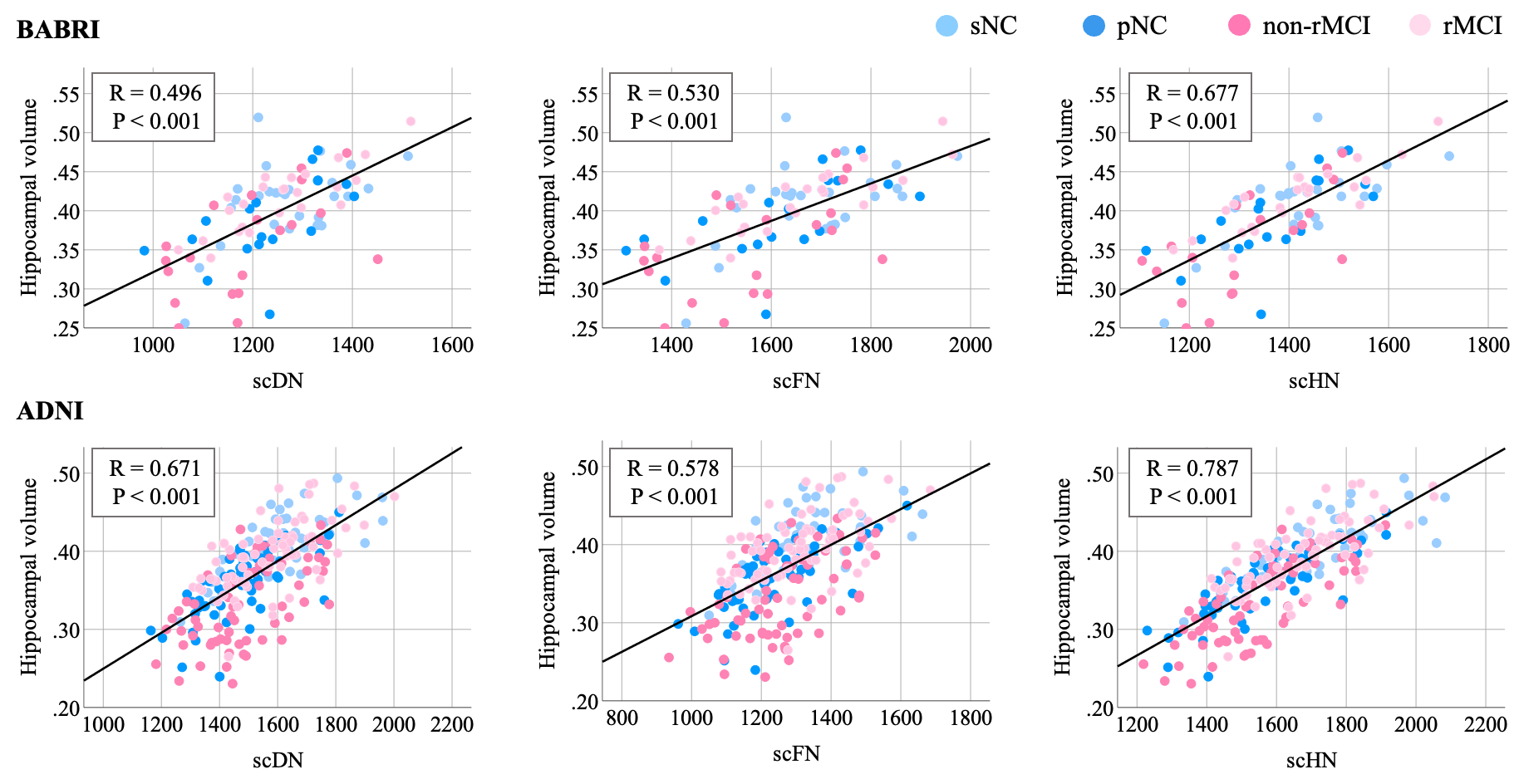


**Fig. S5 The relationship between the composite score of the structural covariance network and the gray matter density of the hippocampus.** Abbreviations: scDN, structural covariance of the default network; scFN, structural covariance of the frontoparietal control network; scHN, structural covariance of the hippocampal network.
